# Detection and treatment of omega-3 fatty acid deficiency in psychiatric practice: Rationale and implementation

**DOI:** 10.1186/s12944-016-0196-5

**Published:** 2016-02-10

**Authors:** Erik Messamore, Robert K. McNamara

**Affiliations:** Department of Psychiatry and Behavioral Neuroscience, University of Cincinnati College of Medicine, 260 Stetson Street, Rm. 3306, Cincinnati, OH 45218-0516 USA; Lindner Center of HOPE, Mason, OH USA

**Keywords:** Long-chain omega-3 fatty acids, Eicosapentaenoic acid (EPA), Docosahexaenoic acid (DHA), Bipolar disorder, Major depressive disorder, Schizophrenia, ADHD

## Abstract

A body of translational evidence has implicated dietary deficiency in long-chain omega-3 (LC*n*-3) fatty acids, including eicosapenaenoic acid (EPA) and docosahexaenoic acid (DHA), in the pathophysiology and potentially etiology of different psychiatric disorders. Case–control studies have consistently observed low erythrocyte (red blood cell) EPA and/or DHA levels in patients with major depressive disorder, bipolar disorder, schizophrenia, and attention deficit hyperactivity disorder. Low erythrocyte EPA + DHA biostatus can be treated with fish oil-based formulations containing preformed EPA + DHA, and extant evidence suggests that fish oil supplementation is safe and well-tolerated and may have therapeutic benefits. These and other data provide a rationale for screening for and treating LC*n*-3 fatty acid deficiency in patients with psychiatric illness. To this end, we have implemented a pilot program that routinely measures blood fatty acid levels in psychiatric patients entering a residential inpatient clinic. To date over 130 blood samples, primarily from patients with treatment-refractory mood or anxiety disorders, have been collected and analyzed. Our initial results indicate that the majority (75 %) of patients exhibit whole blood EPA + DHA levels at ≤4 percent of total fatty acid composition, a rate that is significantly higher than general population norms (25 %). In a sub-set of cases, corrective treatment with fish oil-based products has resulted in improvements in psychiatric symptoms without notable side effects. In view of the urgent need for improvements in conventional treatment algorithms, these preliminary findings provide important support for expanding this approach in routine psychiatric practice.

## Background

Limited efficacy and adverse side-effects associated with conventional psychotropic medications used for the treatment of psychitaric disorders highlight an urgent need to identify modifiable risk and resilience mechanisms to inform improvements in current treatment algorithms. Over the past three decades a substantial body of evidence has implicated a dietary deficiency in long-chain omega-3 (LC*n*-3) fatty acids, eicosapenaenoic acid (EPA) and docosahexaenoic acid (DHA), in the pathophysiology of different recurrent psychiatric disorders, including major depressive disorder (MDD), bipolar disorder, schizophrenia, and attention deficit hyperactivity disorder (ADHD). While this body of evidence has been slow to impact conventional psychiatric training and practice, the field is slowing evolving and nutritional medicine is gaining credibility [1].

This review provides a brief overview of evidence implicating LC*n*-3 fatty acid deficiency in the pathophysiology and potentially etiology or recurrent psychiatric disorders, and then discusses strategies to translate this evidence into clinical screening and treatment algorithms. We present preliminary data from a recently implemented pilot program which routinely measures blood fatty acid levels in psychiatric patients entering a residential inpatient clinic. Four notable cases that illustrate the benefits resulting from detecting and treating LCn-3 fatty acid deficiency are presented.

## LC*n*-3 fatty acids

As background, omega-3 (*n*-3) and omega-6 (*n*-6) fatty acids are members of the polyunsaturated fatty acid (PUFA) family. Prominent dietary sources of the short-chain *n*-3 fatty acid precursor α-linolenic acid (ALA, 18:3*n*-3) include flaxseed, linseed, canola, soy, and perilla oils. Prominent dietary sources of the short-chain *n*-6 fatty acid precursor linoleic acid (18:2*n*-6) include safflower, soy, and corn oils. These PUFAs are considered ‘essential’ because mammals are entirely dependent on dietary sources to procure and maintain adequate concentrations in peripheral and central tissues. Long-chain (LC) fatty acids derived from these short-chain precursors require a series of common and competitive biosynthetic reactions. In human subjects, however, LC fatty acid biosynthesis is extremely inefficient, and dietary intake of preformed LC*n*-3 and LC*n*-6 fatty acids is significantly more effective for increasing peripheral and presumably central levels [2–6]. Preformed LC*n*-3 fatty acids including EPA (20:5*n*-3) and DHA (22:6*n*-3) can be obtained directly from fatty cold water fish, including salmon, trout, tuna, as well as fish oil and algal-derived supplements, and preformed LC*n*-6 fatty acids including arachidonic acid (AA, 20:4*n*-6) can be obtained directly from animal-based foods including beef, chicken, and eggs.

## Animal neurodevelopmental studies

Animal studies have provided important insight into the role of dietary LC*n*-3 fatty acids in the maturation of multiple neuronal systems implicated in the pathoetiology and treatment of psychiatric disorders. The advantage of animal studies is the ability to systematically and selectively manipulate dietary *n*-3 fatty acid intake and to control myriad extraneous variables that frequently confound interpretation of clinical studies. In general, rodent feeding studies have demonstrated that cortical accrual of DHA, the primary LC*n*-3 fatty acid in mammalian brain, during perinatal development is positively associated with cortical neurogenesis [7–9], neuroblast migration [10], neuronal differentiation [11], neurotrophic factor (NGF, BDNF) expression [12, 13], nerve growth factor-induced neurite outgrowth and synaptogenesis [14–17], and synaptic pruning [18]. Cortical DHA deficiency during development is also associated with neuroinflammation [19], and an enduring dysregulation in multiple neurotransmitter systems including dopamine and serotonin which are reversible with early, but not later, postnatal *n*-3 fatty acid supplementation [20, 21]. In addition to neurotrophic and anti-inflammatory effects, DHA and its bioactive metabolites are protective against a variety of insults associated with oxidative stress and lipid peroxidation in the fetal and adult rat brain [22–33]. Behavioral studies suggest that development deficits in brain DHA accrual are associated with elevated behavioral indices of depression that emerge after puberty [34, 35]. In contrast, dietary fish oil fortification significantly decreases depression-like behavior similar to antidepressant medications [36, 37]. These and other data highlight the importance of DHA for normal brain maturation.

In non-human primates, dietary-induced *n*-3 fatty acid deficiency during development is associated with deficits in visual attention [38], polydipsia [39], and deficits in visual acuity and electroretinogram abnormalities [40, 41]. Electroretinogram abnormalities have also been observed in neonatal baboons born preterm [42]. Perinatal *n*-3 fatty acid deficiency is associated increased home cage stereotypy and locomotion bouts [43], which is consistent with dysregulated mesolimbic dopamine activity. A neuroimaging study found that resting-state functional connectivity among prefrontal cortical networks was impaired in monkeys raised on an *n*-3 fatty acid deficient diet compared with monkeys raised on fish oil-fortified diet [44]. Specifically, *n*-3 fatty acid deficiency during perinatal development was associated with reduced resting-state connectivity between the dorsal anterior insula (seed-region) and ventromedial, orbitofrontal, and dorsolateral prefrontal regions, as well as superior temporal and medial parietal regions, compared with monkeys raised on a fish oil-fortified diet. A positron emission tomography study demonstrated that dietary supplementation with DHA improves the decline in neurovascular coupling observed in aged monkeys [45]. As with rodents, these findings suggest that low cortical DHA status is associated with a range of enduring neurodevelopmental abnormalities.

## Human neurodevelopmental studies

During pregnancy DHA accumulates in human neonatal brain tissue at an accelerated rate during the third trimester in association with rapid changes in cortical structural maturation [46, 47]. Prospective longitudinal studies have observed a positive relationship between fetal cord blood DHA levels and neurodevelopmental outcomes in older children [48–54]. As observed in non-human primates [55], human infants born preterm exhibit lower erythrocyte and postmortem cortical DHA concentrations compared with term infants [56–60]. Preterm birth and/or low birth weight is associated with increased risk for developing ADHD in childhood [61–63], and mood, anxiety, and psychotic disorders during adolescence and young adulthood independent of maternal history of psychiatric illness [64–69]. Studies also suggest that longer breastfeeding duration, a putative surrogate for early postnatal DHA intake, is associated with better neurocognitive outcomes [70–72] and decreased risk for ADHD [73–76]. Recent neuroimaging findings suggest that low LC*n*-3 fatty acid intake and biostatus may impair cortical structural and functional maturation in corticolimbic regions repeatedly implicated in psychopathology [77–86]. Together these findings suggest that early and uncorrected deficits in DHA accrual during development are associated with suboptimal brain development and may increase risk of psychopathology emerging in childhood and adolescence.

## Relevance to psychopathology

### Epidemiological evidence

Cross-national epidemiological surveys have observed a significant inverse correlation between per capita fish or seafood consumption (primary dietary sources of preformed EPA + DHA) and lifetime prevalence rates of MDD [87, 88], postpartum depression [89], and bipolar spectrum disorders [90]. While the prevalence rates of schizophrenia appear to be consistent across different countries and not associated with per capita fish or seafood consumption [88], functional outcomes are better in counties with higher per capita intake of vegetables, fish or seafood [91]. There is also large cross-national variation in the prevalence rates of ADHD, with lower rates being observed in Asian countries compared with Western counties [92]. Cross-sectional studies similarly suggest that habitual diets low in LC*n*-3 fatty acids are associated with increased prevalence rates of depressive symptoms in adolescents [93–96]. A retrospective study found that the shift away from fish-based to Western diets in Arctic communities was associated with increased rates of seasonal affective disorder, depression, suicide, and cardiovascular disease [97]. While these findings provide indirect support for an inverse association between fish-based diets and prevalence rates of some psychiatric disorders, most notably depression, myriad variables may also be protective either individually or in combination with fish intake.

Several lines of evidence also suggest that increasing LC*n*-3 fatty acid status may reduce risk of suicide, a primary cause of excess premature mortality in mood and psychotic disorders [98–101]. First, cross-sectional epidemiological surveys have observed an inverse correlation between dietary LC*n*-3 fatty acid intake and the prevalence of suicidal ideation in the general population in Finland [102], and that seasonal variations in violent suicide rates coincide with seasonal variations in serum LC*n*-3 fatty acid levels [103]. In two case–control studies, erythrocyte or plasma LC*n*-3 fatty acid levels were significantly lower in suicidal patients [104, 105], and a prospective surveillance study found that low baseline plasma DHA composition was a significant predictor of future suicidal attempts in medication-free patients with MDD [106]. Two controlled trials found that chronic (12 week) dietary LC*n*-3 fatty acid treatment reduced suicidality in MDD patients [107, 108]. However, prospective cohort studies conducted in the United States (U.S.) have not observed an association between LC*n*-3 fatty acid intake and completed suicide in the general population [109]. Therefore, while extant evidence suggests that increasing LC*n*-3 fatty acid status in patients with psychiatric illness may be protective against suicidality, additional research is needed to evaluate whether depressed mood mediates this effect.

**Fig. 1:**
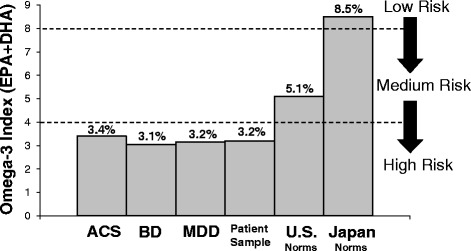
Comparison of mean EPA + DHA levels in adult patients with acute coronary syndrome (ACS) residing in the U.S. (erythrocytes, *n* = 768) [119], first-episode bipolar disorder (BD) (erythrocytes, *n* = 40) [130] and adolescent MDD (erythrocytes, *n* = 20) [134] patients residing in the Cincinnati area, psychiatric patients admitted to the inpatient clinic at The Lindner Center of HOPE, Cincinnati (whole blood, *n* = 131), normative values from a cohort subjects residing in the U.S. (whole blood, *n* = 27,414, http://www.omegaquant.com/fatty-acids-regularly-measured/), and adults residing in Japan (erythrocytes, *n* = 456) [124]. Proposed ‘risk zones’ for sudden cardiac death derived from prospective longitudinal studies are indicated [118]. Note that psychiatric patients exhibit EPA + DHA levels that are similar to patients with ACS and place them at high risk for sudden cardiac arrest. It is proposed that similar ‘risk zones’ be adopted in psychiatric practice to identify patients requiring corrective LC*n*-3 fatty acid supplementation

Excess premature mortality in patients with mood and psychotic disorders is also attributable in part to cardiovascular-related diseases [98–101], and associated risk factors are apparent early in the course of illness (i.e., in adolescents)[110]. Multiple lines of evidence suggest that LC*n*-3 fatty acid deficiency may increase risk for cardiovascular disease morbidity and mortality [111]. Cross-national epidemiological studies have observed reduced prevalence rates of coronary heart disease morbidity and mortality in populations whose habitual diets include fish [112]. Fatty acid composition studies have found an inverse correlation between erythrocyte EPA + DHA levels and cardiovascular risk factors including elevated serum triglyceride and pro-inflammatory molecules [113, 114]. LC*n*-3 fatty acid supplementation is efficacious for the treatment of elevated serum triglyceride levels, an independent risk factor for cardiovascular disease, and prescription fish oil-based products have been approved by the U.S. Food and Drug Administration (FDA) for the treatment of hypertriglyceridemia [115]. Prospective studies suggest that low baseline erythrocyte EPA + DHA (‘omega-3 index’) levels are associated with increased risk of sudden cardiac arrest [116–118]. It is relevant, therefore, that the low erythrocyte EPA + DHA levels observed in patients with mood and psychotic disorders are similar to those observed in patients suffering acute coronary syndrome [119], and would be anticipated to increase their risk for sudden cardiac arrest (Fig. 1). While the effects of LC*n*-3 fatty acid supplementation on cardiovascular events and sudden cardiac arrest in patients with a history of cardiovascular disease have been equivocal [120], primary prevention studies in subjects without a history of cardiovascular disease [121] or subjects at high-risk for cardiovascular disease [122] suggest that increasing LC*n*-3 fatty acid biostatus may have protective benefits. While there have been no LC*n*-3 fatty acid primary prevention studies conducted in patients with mood or psychotic disorders, existing evidence provides a rationale for identifying and treating LC*n*-3 fatty acid deficiency in psychiatric patients, particularly those presenting with cardiovascular risk factors.

## Biological evidence

Cross-national habitual dietary fish intake is positively correlated with erythrocyte membrane EPA + DHA composition [123, 124], and fish oil supplementation increases erythrocyte EPA + DHA composition in a linear dose-dependent manner [125]. Several independent case–control studies from different countries have investigated erythrocyte and/or plasma phospholipid EPA + DHA levels in patients with psychiatric disorders. A meta-analysis of 14 case–control studies found significant deficits in EPA and DHA in plasma and erythrocytes from MDD patients [126]. In bipolar patients, four studies observed significant erythrocyte DHA and/or EPA deficits compared with healthy controls [127–130]. Cross-sectional studies have similarly found that pediatric and adolescent patients with or at ultra-high risk for mood disorders exhibit erythrocyte EPA + DHA deficits compared with healthy youth [131–134]. Robust erythrocyte EPA + DHA deficits were also observed in non-depressed patients with social anxiety disorder [135], and plasma EPA + DHA deficits were observed in MDD patients with comorbid anxiety disorders [136]. Medication-naïve first-episode psychotic patients exhibit erythrocyte DHA and AA deficits compared with healthy controls [137–139], and a recent meta-analysis of 18 case–control studies observed significant DHA and AA deficits in schizophrenic patients [140]. A recent meta-analysis of nine cross-sectional studies observed significantly lower blood EPA + DHA levels in ADHD children compared with healthy controls [141]. Together, these studies provide strong evidence that different psychiatric disorders are characterized by low blood DHA and/or EPA biostatus which coincides with, and may precede, the initial onset of psychopathology.

Adult human erythrocyte and frontal cortex DHA levels are positively correlated [142], and a growing number of case–control studies have investigated the fatty acid composition of postmortem frontal gray matter from patients with psychiatric disorders. Some studies have observed lower LC*n*-3 fatty acid levels [143–147] while others have not [148–150]. Limitations associated with the postmortem approach may contribute to discrepant results [151]. Nevertheless, emerging evidence from neuroimaging studies provide support for a positive association between LC*n*-3 fatty acid intake or blood levels on cortical structural and functional integrity over the lifespan [77–86]. For example, greater habitual dietary LC*n*-3 fatty acid intake, which is positively correlated with erythrocyte EPA + DHA composition, was associated with larger cortical gray matter volumes in several corticolimbic regions including the anterior cingulate cortex, hippocampus, and amygdala [83]. It is relevant, therefore, that patients with psychiatric disorders commonly exhibit gray matter volume deficits in the anterior cingulate cortex, hippocampus, and amygdala [152–154]. Moreover, a recent study found that fish oil supplementation increased white matter microstructural integrity in MDD patients in association with reductions in depression symptom severity [155]. Together these findings suggest that there may be a link between low LC*n*-3 fatty acid biostatus and deficits in corticolimbic structural and functional integrity observed in patients with psychiatric disorders.

## LC*n*-3 fatty acid supplementation

Dietary LC*n*-3 fatty acid supplementation has been found to significantly increase erythrocyte LC*n*-3 fatty acid levels in different patient populations [138, 156–158]. This observation indicates that LC*n*-3 fatty acid deficits in psychiatric patients are modifiable by increasing dietary LC*n*-3 fatty acid intake. Meta-analyses of controlled trials suggest that LC*n*-3 fatty acid supplementation may be effective for reducing attention deficits in pediatric and adolescent ADHD patients [141, 159]. Accumulating evidence suggests that LC*n*-3 fatty acid supplementation may be efficacious for the treatment of positive and negative symptoms in patients with or at ultra-high risk for developing schizophrenia [156, 160, 161], and for preventing or delaying the onset of psychosis in ultra-high risk youth [156]. A recent meta-analysis of controlled trials suggests that LC*n*-3 fatty acids may be efficacious during the prodromal stage and in first-episode psychotic patients but not in patients with chronic schizophrenia [162]. These data suggest that early correction of LC*n*-3 fatty acid deficits may have greater efficacy compared with treatment initiated following chronic illness.

Preliminary trials have found that LC*n*-3 fatty acid supplementation, either as monotherapy or adjunctive treatment, significantly reduces depression and manic symptom severity in pediatric and adolescent patients [134, 157, 158, 163]. Meta-analyses of controlled trials have observed a significant advantage of LC*n*-3 fatty acid supplementation over placebo for reducing depressive symptoms in patients with MDD [164, 165] or bipolar disorder [166]. A recent meta-analysis conducted on controlled trials employing exclusively adult MDD patients observed a small-modest benefit for reducing depressive symptoms [167]. In the latter study, the authors concluded that this effect was unlikely to be clinically meaningful and that existing evidence was low quality. Indeed, heterogeneity in study design in terms of daily dose, EPA:DHA ratio, trial duration, concomitant psychotropic medications, use of a bioactive placebo (i.e., olive oil), sample size, and baseline symptom severity likely contribute substantial variability. It is also important to recognize that neurostructural and neurochemical perturbations resulting from LC*n*-3 fatty acid deficiency during development may not be reversible with short-term supplementation. This is supported by rodent studies finding that enduring impairments in serotonin and dopamine neurotransmission resulting from perinatal *n*-3 fatty acid deficiency are reversible with early but not later *n*-3 fatty acid supplementation despite normalization of LC*n*-3 fatty acid biostatus [20, 21]. Therefore, detection and treatment of LC*n*-3 fatty acid deficiency early in the course of illness may be required to exert the greatest protection against recurrent depressive symptoms.

In many controlled LC*n*-3 fatty acid intervention studies observing benefits for depressive symptoms, patients were also receiving conventional antidepressant medications. This suggests that adjunctive LC*n*-3 fatty acid treatment may augment the therapeutic efficacy of antidepressant medications. This is directly supported by studies comparing selective serotonin reuptake inhibitor (SSRI) treatment with or without adjunctive LC*n*-3 fatty acids in patients with MDD. In these studies, adjunctive LC*n*-3 fatty acid treatment augmented the therapeutic efficacy of fluoxetine [168] or citalopram [169]. Additionally, adjunctive LC*n*-3 fatty acid treatment was found to reduce depression symptom severity in adolescent and adult MDD patients that were refractory to standard antidepressant treatment [108, 134]. Adjunctive LC*n*-3 fatty acid treatment was also found to reduce relapse rates in predominantly medicated adult patients with bipolar disorder [170], and to reduce manic symptom severity in medicated pediatric patients with bipolar disorder [158]. In patients with first-episode psychosis, adjunctive LC*n*-3 fatty acid (EPA) treatment accelerated treatment response, improved tolerability, and permited a 20 percent reduction in second-generation antipsychotic medication dose [171]. In addition to augmenting efficacy, adjunctive LC*n*-3 fatty acid treatment may also be protective against adverse cardiometabolic [172–174] and hepatic [175–177] side-effects associated with second generation antipsychotic medications. These preliminary findings suggest that adjunctive LC*n*-3 fatty acid treatment may augment therapeutic efficacy and mitigate cardiometabolic side-effects associated with conventional psychotropic medications.

## Implementation

In the previous sections we briefly summarized a body of translational evidence which suggests that LC*n*-3 fatty acid deficiency, particularly during perinatal development, may represent a modifiable risk factor for psychopathology. Among these findings, meta-analyses of independent case–control studies demonstrate that patients with different psychiatric disorders, including mood, psychotic, and attentional disorders, exhibit significantly lower blood levels of EPA and/or DHA compared with healthy demographically similar controls. Additionally, meta-analyses of several independent controlled intervention studies suggest that increasing EPA + DHA biostatus via dietary supplementation with fish oil-based products may be effective for reducing psychiatric symptoms, suicidality, and cardiometabolic risk factors. Together these and other data support routine screening for and treatment of LC*n*-3 fatty acid deficiency in patients with psychiatric disorders. Below we briefly discuss existing screening and treatment resources and general guidelines required for implementation in psychiatric practice.

## Diagnosing LC*n*-3 fatty acid deficiency

There are currently several Clinical Laboratory Improvement Amendments (CLIA)-certified laboratory facilities that routinely perform blood fatty acid analyses by gas–liquid chromatography (GLC). While the American Medical Association has several current procedural terminology (CPT) codes for blood fatty acid analysis by GLC (82725, 82544, 82492) and GLC testing in general (82541, 82542), reimbursement for these tests is ultimately at the discretion of the insurance provider. For this procedure, whole blood (~25 uL) is obtained from a finger prick and is spotted and dried onto anti-oxidant treated card which is then shipped at ambient temperature. The GLC analysis involves the determination of different fatty acids including EPA + DHA which are reported as a percentage of total fatty acids. Fatty acid levels are returned to the physician in approximately 2 weeks. Individual fatty acid levels can be compared to U.S. population norms. Against this reference, a patient’s fatty acid levels can be expressed in a percentile ranking. Based on evidence that erythrocyte EPA + DHA composition of ≤4 % of total fatty acid composition increases risk for sudden cardiac death [118], and evidence that the majority of patients with psychiatric disorders exhibit erythrocyte EPA + DHA composition of ≤4 % [128, 130, 132, 134, 178], erythrocyte or whole blood EPA + DHA composition of ≤4 % may be considered to be a ‘state of deficiency’ that requires corrective intervention. Analogous to routine cholesterol testing, this screening approach can provide a valid, reliable, and relatively non-invasive measure of a patient’s EPA + DHA biostatus.

## Treating LC*n*-3 fatty acid deficiency

Prescription ethyl ester EPA + DHA (Lovaza® in the US, Omacor® in Europe, GlaxoSmithKline), purified ethyl ester EPA containing no DHA (Vascepa®, Amarin Corporation), and a free versus ethyl ester EPA + DHA formulation (Epanova®, AstraZeneca) have been approved by the U.S. FDA for the treatment of hypertriglyceridemia (≥500 mg/dL). More recently a generic version of Lovaza has become available (Teva Pharmaceuticals USA, Inc.). Over-the-counter fish oil supplements containing similar ethyl ester EPA + DHA concentrations are also widely available. It is important to note, however, that no LC*n*-3 fatty acid formulation is currently approved by the FDA for the treatment of any psychiatric disorder.

The American Psychiatric Association has adopted the consensus recommendations of the American Heart Association for an EPA + DHA dose of 1 g/d in patients with MDD [179]. The American Heart Association also recommends 3 g/d EPA + DHA for reducing elevated triglyceride levels. Controlled intervention studies have in general found that daily doses in the range of 0.2–2 g/d of EPA + DHA may be efficacious for the treatment of mood symptoms [164, 165]. Emerging evidence also suggests that a larger ratio of EPA to DHA may be more efficacious for treating depressive symptoms [165] as well as ADHD symptoms [159]. A dose–response study found that EPA + DHA supplementation at doses between 300–900 mg/d are sufficient to increase erythrocyte EPA + DHA composition in non-psychiatric subjects to levels thought to be cardioprotective [125]. As with other medications, upward dose titration may be required as clinically indicated. For example, in an open-label flexible dosing study 8-week LC*n*-3 fatty acid *monotherapy* led to a statistically significant reduction in manic symptom severity scores in pediatric bipolar patients [157]. In this study the starting dose was 1.3 g/d of EPA + DHA, the maximum dose was 4.3 g/d, and the mean dose was 2.6 g/d. While there is a need for additional dose-titrating secondary prevention trials to elucidate optimal LC*n*-3 fatty acid dosing strategies to maximize therapeutic effects, existing evidence suggests that EPA + DHA doses in the range of 1–4 g/d are sufficient to treat erythrocyte EPA + DHA deficits in patients with psychiatric illness.

The U.S. Food and Drug Administration (FDA) considers LC*n*-3 fatty acid doses up to 3 g/d to be ‘generally regarded as safe’. Potential adverse events associated with LC*n*-3 fatty acid treatment include gastrointestinal disturbances, including nausea, diarrhea, gastroesophageal reflux, eructation, and less commonly emesis. In double-blind clinical trials in adolescent and adult patients, the principal adverse events reported after chronic (8–12 weeks) treatment with LC*n*-3 fatty acid supplements were gastrointestinal problems, and were considered mild and not clinically significant [134, 158, 163, 179]. To minimize the gastrointestinal adverse events associated with LC*n*-3 fatty acids, patients should be instructed to take their pills with meals. Although taking fish oil at high doses (>3 g/d) has been associated in isolated cases with increased bleeding time in subjects also taking anticoagulant medications [180], controlled clinical trials have found that chronic high dose EPA + DHA alone or in combination with aspirin does not increase risk for clinically-significant increases in bleeding time [181–183]. Another safety consideration involves the potential threat of contamination of fish and seafood with methyl mercury and other environmental pollutants. However, most fish oil supplements are highly purified and do not exceed U.S. FDA limits for methyl mercury and other environmental contaminants. As with all medications, patients should be informed of potential risks associated with fish oil-based products, and patients with an allergy to shellfish or seafood should be closely monitored.

## Proof-of-concept

The Lindner Center of HOPE (http://www.lindnercenterofhope.org) is a university-affiliated mental health care center located in Mason, a suburb of Cincinnati, Ohio. The center houses an outpatient mental health clinic, acute care hospital services, and specialized residential diagnostic assessment programs. The residential diagnostic assessment programs serve approximately 280 pediatric, adolescent, and adult patients annually. Patients are typically referred to these programs by mental health clinicians when there is diagnostic uncertainty, and multiple treatment failures is the most frequent cause of diagnostic uncertainty. Complex affective or anxiety disorders are the most common reasons for referral. Diagnostic assessment within these programs includes medical examination, neurological examination, laboratory studies, brain imaging or EEG when indicated, neuropsychological testing, objective personality assessment, structured diagnostic interview, serial mental status evaluations, continuous behavioral observations in the residential milieu, and acquisition of collateral behavioral histories from families, close associates, or outpatient healthcare providers.

**Fig. 2:**
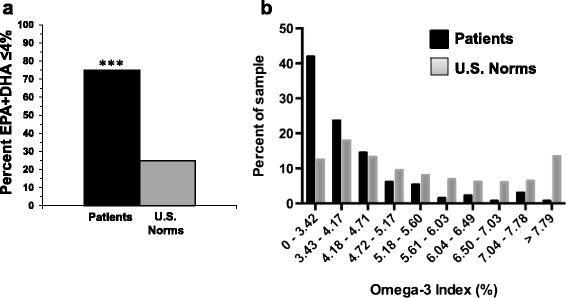
**a** The percentage of subjects residing in the U.S. (*n* = 27,426) and psychiatric patients admitted to the inpatient clinic at The Lindner Center of HOPE (*n* = 131) with an ‘omega-3 index’ (whole blood EPA + DHA) of ≤4.0 % of total fatty acid composition. **b** Histogram comparing ‘omega-3 index’ (EPA + DHA) frequency distribution of the psychiatric patient sample to the OmegaQuant reference sample. Note that the majority (75 %) of psychiatric patients exhibit whole blood EPA + DHA levels at ≤4 percent of total fatty acid composition, a rate that is significantly greater than general U.S. population norms (25 %, ***P ≤ 0.0001, Chi-Square)

Beginning in mid-2014, measurement of whole blood fatty acid levels was implimented as part of the routine laboratory assessment. Dried blood spots are sent to OmegaQuant, LLC for analysis. To date over 131 patient blood samples have been collected and analyzed. The majority of these patients were referred for mood or anxiety disorders and had previously responded poorly to standard outpatient care (i.e., treatment-refractory). Our initial results suggest that the majority (75 %) of patients exhibit whole blood EPA + DHA levels at ≤4 percent of total fatty acid composition. This rate is significantly greater than general U.S. population norms (25 %) based on 27,414 subjects residing in the U.S. (p ≤ 0.0001) (Fig. 2a). The overall (*n* = 131) EPA + DHA mean for patients is 3.2 % of total fatty acid composition, which is at the 8th percentile of fatty acid norms and lower than the mean observed for the general U.S. population (5.1 %, 25th percentile) (Fig. 2b). It is notable that the mean EPA + DHA level observed in psychiatric patients is similar to that observed in erythrocytes from U.S. adult patients suffering acute coronary syndrome (3.4 %)[119] (Fig. 1), which places them at elevated risk for developing sudden cardiac arrest [118]. Our initial results therefore support prior evidence that psychiatric patients exhibit very low EPA + DHA levels. Below we describe four notable cases that illustrate the benefits resulting from detecting and treating LC*n*-3 fatty acid deficiency.

## Clinical vignettes

### Case 1

A 24 year old man with childhood onset of psychiatric symptoms, at least 4 prior psychiatric hospitalizations, and historical diagnoses of type 2 bipolar disorder, attention deficit-hyperactivity disorder, eating disorder, and borderline personality disorder was found to have an omega-3 index (EPA + DHA) of 2.98 % which placed him at the 4th percentile of U.S. population norms. Based on this, he was prescribed Lovaza (ethyl-EPA/ethyl-DHA) at a daily dose of 1,860 mg EPA and 1,500 mg DHA (total EPA + DHA dose: 3,360 mg/d) which was covered by his insurer. Based on his history of bipolar disorder, his divalproex prescription was continued. However, based on lack of efficacy his vilazadone and lisdexamphetamine were discontinued. Within three weeks the patient reported to his outpatient psychiatrist that his mental status, mood and mood stability, and overall level of social and occupational functioning were the best he could ever recall. At 6-month follow-up his omega-3 index (EPA + DHA) had risen to 7.69 % (85th percentile), and he reported that he was enjoying a continued stability previously unknown to him. He credited his success to the addition of the ethyl-EPA/ethyl-DHA, stating that he had never had such benefit associated with divalproex. He also discounted the possibility that discontinuation of his antidepressant and stimulant medications were relevant, since there had been many prior episodes of being off these medications with no recalled equal benefit.

### Case 2

A 27 year old woman was admitted with a chief complaint of emotional dysregulation. Although there was a family history of bipolar disorder, the patient had no history of manic episodes; however, she did engage in frequent suicidal gestures and self-injurious behaviors. The patient also had experienced simple partial seizures since early childhood. Her omega-3 index (EPA + DHA) was 2.93 % of total fatty acids (3rd percentile). Based on the family history of bipolar disorder, lithium was added to her existing medications. Her topiramate and oxcarbazapine were continued. Based on the low level of omega-3 fatty acids, she was also started on Lovaza (ethyl-EPA/ethyl-DHA) at a dose of 1860/1500 mg daily (total EPA + DHA: 3,360 mg/d). Consistent with recent evidence that fish oil supplementation elevates seizure thresholds and reduces seizure frequency in epileptic patients [184, 185], at six-month follow-up she had been seizure-free, was able to live independently and reported no self-injurious behaviors.

### Case 3

A 23 year old man was referred for treatment because he was contemplating suicide because he could no longer tolerate near-constant depression and anxiety. He had suffered from severe anxiety since age 5. He had been treated with a wide variety of antidepressant medications, mood stabilizers, and stimulants since his early teens. Medications had been either ineffective or poorly tolerated. He was diagnostically enigmatic and over the course of his life had been diagnosed with anxiety disorder, major depression, bipolar disorder, and attention deficit hyperactivity disorder. Comprehensive diagnostic assessment suggested generalized anxiety disorder and bipolar disorder not otherwise specified as the most appropriate diagnoses. Fatty acids analysis revealed an omega-3 index of 3.38 % (11th percentile). Because of this, addition of omega-3 fatty acids to his medications was recommended. He took an over-the-counter fish oil supplement at a dose that delivered 1600 mg of EPA and 800 mg of DHA per day (total EPA + DHA: 2,400 mg/d). Other treatment recommendations included: discontinuation of serotonin reuptake inhibitors, addition of lithium, and addition of a low dose of topiramate (50 mg nightly). He was continued on his existing 200 mg nightly dose of quetiapine. At nine-month follow-up, he reported markedly improved social and occupational functioning and normalization of his mood. The durability of his improvements allowed for elimination of lithium and topiramate and halving of his quetiapine dose. He continued to take the EPA+DHA supplement.

### Case 4

A 38 year old woman reported constitutively high anxiety levels since childhood and also described highly reactive mood and difficulty managing anger, and identified anxiety as her most pressing concern. She had tried numerous serotonin reuptake inhibitor medications with no discernable benefit. She reported a history of problematic use of alcohol and benzodiazepines, though she had not used either substance for several months prior to her assessment. She was diagnosed with anxiety disorder, not otherwise specified; benzodiazepine abuse in early full remission; and alcohol abuse in early full remission. Borderline personality traits were prominent, but criteria for a personality disorder diagnosis were not fully met. Fatty acid analysis revealed an omega-3 index of 3.25 % (8th percentile). Because serotonergic medications had been historically unhelpful, she was prescribed topiramate to address mood reactivity and anxiety, and to promote sustained abstinence from alcohol, and fish oil supplementation was recommended. She elected to take an over-the-counter fish oil supplement at a dose that delivered an EPA dose of 1080 mg/d and DHA dose of 720 mg/d (total EPA + DHA: 1,800 mg/d). At three month follow-up, she reported adherence to recommended dialectical behavior therapy and had continued to take topiramate (50 mg twice daily) and the fish oil supplement. She reported significant and sustained reduction of anxiety, improved mood stability, abstinence from alcohol or benzodiazepines, and improved social and interpersonal functioning.

## Conclusions

There is now a substantial body of translational evidence that suggests that LC*n*-3 fatty acid deficiency, particularly during active periods of brain development, is associated with neuropathogenic features that have separately been implicated in the pathophysiology of different psychiatric disorders. Moreover, major recurrent psychiatric disorders are associated with blood LC*n*-3 fatty acid deficiency which may increase risk for cardiovascular disease, a principle cause of excess premature mortality in patients with mood and psychotic disorders. Extant evidence suggests that LC*n*-3 fatty acid supplementation is sufficient to increase LC*n*-3 fatty acid blood levels in psychiatric patients which may additionally have therapeutic benefits and mitigate adverse cardiometabolic risk factors. These and other data provide a strong rationale supporting routine screening for and treatment of LC*n*-3 fatty acid deficiency in patients with psychiatric illness. As proof-of-concept, we have demonstrated the feasibility of implementing blood LC*n*-3 fatty acid screening as well as treatment using fish oil-based formulations in a residential diagnostic assessment center. Consistent with prior cross-sectional evidence, our preliminary results demonstrate that the majority of these patients exhibit very low blood EPA + DHA levels, and that treatment with fish oil can result in improvements in psychiatric symptoms without any notable side effects. Within the context of extant evidence and the urgent need for improvements in conventional treatment algorithms, these preliminary findings provide important support for expanding this approach in routine psychiatric clinical practice.
